# A Biomimetic Sensor for the Classification of Honeys of Different Floral Origin and the Detection of Adulteration

**DOI:** 10.3390/s110807799

**Published:** 2011-08-09

**Authors:** Ammar Zakaria, Ali Yeon Md Shakaff, Maz Jamilah Masnan, Mohd Noor Ahmad, Abdul Hamid Adom, Mahmad Nor Jaafar, Supri A. Ghani, Abu Hassan Abdullah, Abdul Hallis Abdul Aziz, Latifah Munirah Kamarudin, Norazian Subari, Nazifah Ahmad Fikri

**Affiliations:** Sensor Technology and Applications Group (STAG), Universiti Malaysia Perlis (UniMAP), 01000, Kangar, Perlis, Malaysia; E-Mails: aliyeon@unimap.edu.my (A.Y.M.S.); mazjamilah@unimap.edu.my (M.J.M.); mohdnoor@unimap.edu.my (M.N.A.); abdhamid@unimap.edu.my (A.H.A.); mahmad@unimap.edu.my (M.N.J.); supri@unimap.edu.my (S.A.G.); abuhassan@unimap.edu.my (A.H.A.); abdhallis@unimap.edu.my (A.H.A.Z); munirahkamarudin@gmail.com (L.M.K.); aziansubari@gmail.com (N.S.); naffe_five@yahoo.com (N.A.F.)

**Keywords:** electronic nose, electronic tongue, honey classification, bio-mimicking sensor, floral origin and adulteration

## Abstract

The major compounds in honey are carbohydrates such as monosaccharides and disaccharides. The same compounds are found in cane-sugar concentrates. Unfortunately when sugar concentrate is added to honey, laboratory assessments are found to be ineffective in detecting this adulteration. Unlike tracing heavy metals in honey, sugar adulterated honey is much trickier and harder to detect, and traditionally it has been very challenging to come up with a suitable method to prove the presence of adulterants in honey products. This paper proposes a combination of array sensing and multi-modality sensor fusion that can effectively discriminate the samples not only based on the compounds present in the sample but also mimic the way humans perceive flavours and aromas. Conversely, analytical instruments are based on chemical separations which may alter the properties of the volatiles or flavours of a particular honey. The present work is focused on classifying 18 samples of different honeys, sugar syrups and adulterated samples using data fusion of electronic nose (e-nose) and electronic tongue (e-tongue) measurements. Each group of samples was evaluated separately by the e-nose and e-tongue. Principal Component Analysis (PCA) and Linear Discriminant Analysis (LDA) were able to separately discriminate monofloral honey from sugar syrup, and polyfloral honey from sugar and adulterated samples using the e-nose and e-tongue. The e-nose was observed to give better separation compared to e-tongue assessment, particularly when LDA was applied. However, when all samples were combined in one classification analysis, neither PCA nor LDA were able to discriminate between honeys of different floral origins, sugar syrup and adulterated samples. By applying a sensor fusion technique, the classification for the 18 different samples was improved. Significant improvement was observed using PCA, while LDA not only improved the discrimination but also gave better classification. An improvement in performance was also observed using a Probabilistic Neural Network classifier when the e-nose and e-tongue data were fused.

## 1. Introduction

A large number of Asian countries are highly dependent on their agricultural sectors. Rapid growth of the agro-based industry and the lack of quality assessment have become a cause for concern. The agro-based industry covers a broad spectrum of products, ranging from fresh farm produce to processed foods, herbal products and beverages. Malaysia is a tropical country rich in natural forest resources such as herbs, medicinal plants, spices and honey. These traditional foods are one of the main sources of income for the Malaysian agricultural industry, but unfortunately, some of these traditional products, especially those produced by small scale industries, have not been screened or undergone strict quality assessments.

Current quality assessment or screening methods using analytical instruments are generally time consuming and often operator dependant. With the limited number of testing laboratories available, such assessments are unable to meet the demand of the increasing number of these traditional products.

Furthermore, the quality assessments are essentially still best carried out by human panels due to the subjectivity involved [1]. However, due to the many inherent weaknesses of panel tests, researchers are proposing multi-modality sensing to assist human panels in making their decisions. The aim of this concept is to partially emulate the human sensory systems (*i.e*., smell and taste) electronically and combine them in a similar manner to how they work in the human brain [2,3]. This multi-modality sensor fusion has been accepted in a wide range of specific applications such as the military, medicine and agriculture [4–10]. The advantages of this concept compared to a single modality sensor have been clearly proven [5,6].

Quality assessment of honey is often related to its flavour (taste and aroma), besides its phytochemical contents and nutritional values. Unfortunately, laboratory assessments such as Fourier Transform Infrared Spectroscopy (FTIR), Liquid Chromatography-Mass Spectrometry (LC-MS) and Gas Chromatography-Mass Spectroscopy (GC-MS) are limited to chemical separation [11–17]. Furthermore, it has been reported that analytical instruments were often unable to correlate with the human perception [18–20]. This paper presents two bio-mimicking sensing modalities, namely the electronic nose (e-nose) and electronic tongue (e-tongue). These bio-mimicking sensors are combined by an efficient data fusion algorithm to perform classification of honeys of different floral origin and adulteration. The combination of these electronic sensory inputs allows the possibility of associating the chemical contents with the senses of taste and smell [18].

## 2. Materials and Methods

### 2.1. Sample Selection

In this experiment, five samples of each honey type were prepared from 14 different brands of honey, two different brands of syrup and two different brands of honey mixed with syrups. These 80 samples (5 g each) of honey and syrup were obtained from commercial sources, while another ten samples were freshly prepared by mixing 3 g of different Tualang honeys and 2 g of different syrups. The mixed samples (labeled as M1 and M2) were used as the adulterated samples. In total, a total of 90 samples of honey, syrup and adulterated honey were prepared for this experiment, as summarized in Table 1.

### 2.2. Biochemical Measurements

#### 2.2.1. Fourier Transform Infrared Spectroscopy (FTIR) Measurement

The FTIR spectral measurements were performed at room temperature at 27 °C using a Perkin Elmer 1,600 FTIR Spectrometer (Waltham, MA, USA). This FTIR spectrometer is equipped with an ATR crystal having coverage of 4,000 to 650 cm^−1^ spectral region. The spectral measurements were performed against a background baseline of distilled water and presented in total attenuation units. The crystal surface was cleaned with distilled water and dried with tissue paper (Kimberly-Clark, Malaysia) after the measurement of each sample. The background spectrum was obtained before each sample measurement and verified to ensure the surface of the crystal was clean and free from previous sample residue. A small drop of honey sample was placed on the crystal using a syringe. The measurements of each sample were repeated three times and averaged. The spectra were collected and converted into an ASCII file to be further analysed using MATLAB (ver. 7.0).

#### 2.2.2. Gas Chromatography Mass Spectroscopy (GC-MS) Measurement

Five mL of each sample was added directly into a 22 mL headspace vial and sealed with a PTFE septum. Each vial was placed inside Turbo Matrix HS 16 (HS) and run under headspace mode. The headspace program was set using the TurboMatrix touch screen control system. The carrier gas was set to 20 psi. The HS oven was set to 60 °C and the each vial was preheated for 10 min. The needle and transfer line temperature were set to 65 °C and 70 °C respectively. A de-activated fused silica transfer line connected the HS to the Clarus 680 GC, which was equipped with a programmable split/splitless (PSS) injector, Elite-5MS-30M column (N9316282) and programmable pneumatic control (PPC). The Clarus 600 MS was controlled via TurboMass™ 5.4.2 GC/MS software and operated in electron ionization (EI) mode. The initial oven temperature was programmed to start at 40 °C and held for 5 min. It was then ramped to 150 °C at a rate of 5 °C/min and held for 2 min. The second ramp was started at the rate of 20 °C/min to 280 °C and kept for 30 min. The GC setting strictly followed a standard technique [21,22]. Helium was used as carrier gas and set to 1 mL/min. The MS scan time was set to run for 65 min and the mass scanning range set from *m/z* 20 to 550.0. The scan time was set to 0.2 s with a 0.1 s interscan delay. The headspace compound identification was done by looking at the retention time and comparing with the known library standards and search hits.

#### 2.2.3. pH and Brix Level

The honey, sugar and adulterated samples were analysed for total soluble solids (TSS; °brix), reflective index and pH level using a digital refractometer (Reichert–AR200 Depew, NY, USA) and pH-meter (TESTO 206-pH2 Sparta, NJ, USA), respectively. The brix level in the honey samples was calibrated against distilled water. Both measurements were set with automatic temperature correction and each measurement was repeated for at least three times and the average was obtained. All samples for the brix and reflective index measurements were used without diluting, while for pH measurements, a 20% (w/v) solution of honey with distilled water was prepared for the measurement. Acquarone and Dias [23,24], suggested a suitable dilution of honey for pH measurement should be around 10% to 100% (w/v).

### 2.3. E-Nose Measurements

The Cyranose320 e-nose from Smith Detection™ which uses 32 non-selective sensors of different types of polymer matrix, blended with carbon black was employed. The combination of these 32 sensors as an array allows qualitative and maybe even quantitative assessments of complex solutions [19,25,26]. Persaud [27] have demonstrated that the use of such sensor arrays, together with suitable pattern recognition algorithms can mimic the human olfaction system.

**Figure 1.**
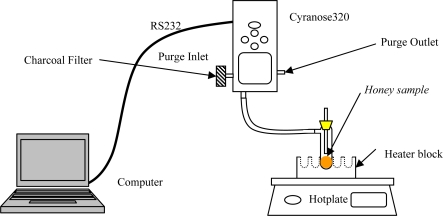
E-nose setup for headspace evaluation of honey, sugar concentration and adulteration sample.

The e-nose setup for this experiment is illustrated in Figure 1 and the settings for the sniffing cycle are also indicated in Table 2. Each sample was drawn from the bottle using a 10 mL syringe and kept in a 13 × 100 mm test tube and sealed with a silicone stopper. Each sample was replicated five times. Before measurement, each sample was placed in a heater block and heated up for 10 min to generate sufficient headspace volatiles. The temperature of the sample was controlled at 60 °C during the headspace collection. Preliminary experiments were performed to determine the optimal experimental setup for the purging, baseline purge and sample draw durations. Ten seconds baseline purge with 30 s sample draw produced an optimal result (result not shown). Baseline purge was set longer to ensure residual gases were properly removed since all the samples were in a liquid form and contained moisture. The pump setting was set to the medium speed during sample draw. The filter used was made up of activated carbon granules and has large surface area which was effective in removing a wide range of volatile organic compounds and moisture in the ambient air. The experiment was carried out using e-nose on a variety of honey samples followed by syrup and adulterated samples.

### 2.4. E-Tongue Measurement

**Figure 2.**
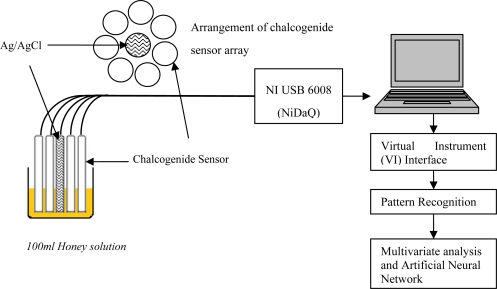
E-tongue setup for headspace evaluation of honey, sugar concentration and adulteration sample.

The chalcogenide-based potentiometric e-tongue was made up of seven distinct ion-selective sensors from Sensor Systems (St. Petersburg, Russia). The same principle explained in Section 2.3 for the e-nose was adopted for the e-tongue to discriminate the complex solutions. Recently, quite a number of successful applications based on the e-tongue assessments were reported [28–33]. Table 3 describes the potentiometric sensors used in this experiment. The e-tongue system shown in Figure 2 was implemented by arranging an array of potentiometric sensors around the reference probe. Each sensor output was connected to the analogue input of a data acquisition board (NI USB-6008) from National Instruments (Austin, TX, USA).

A 5% (w/v) solution of honey in distilled water was prepared and stirred for 3 min at 1,000 rpm before making any measurements. Each sample was replicated five times. For each measurement, the e-tongue was steeped simultaneously and left for five min, and the potential readings were recorded for the whole duration. After each sampling, the e-tongue was dipped for one min in 10% ethanol, stirred at 400 rpm and rinsed twice using distilled water (stirred at 400 rpm for 2 min) to remove any sticky residues from previous samples sticking on the sensor surface to avoid contaminating the next sample.

### 2.5. Data Analysis

The fractional measurement method is essential when using a multi-modalities sensor fusion. This technique is often known as baseline manipulation and was applied to preprocess the data of both modalities [34]. The maximum sensor response, S_t_ is subtracted from the baseline, S_0_ and then divided again by the S_0_. The formula for this dimensionless and normalized *S_frac_*, is determined as follows:

(1) $$S_{\mathrm{frac}}=[S_{t}-S_{0}]/S_{0}$$

This gives a unit response for each sensor array output with respect to the baseline, which compensates for sensors that have intrinsically large varying response levels [35]. It can also further minimize the effect of any temperature, humidity and temporal drifts [35].

The data from different modalities were processed separately and all sensors were used in this analysis. In the case of the e-nose, S_0_ is the minimum value taken during the baseline purge with ambient air and S_t_ was measured during the sample draw. Each sampling cycle was repeated three times and the average was obtained for the five replicated samples. For the e-tongue measurements, S_0_ (baseline reading) is the average reading of distilled water, while S_t_ is the sensor reading when steeped in the solution. The steeping cycle was repeated three times for each sample and the average was obtained for each five of the replicated samples.

Each *S_frac_* data point from each e-nose and e-tongue sensor formed the *S_frac_* matrix. This *S_frac_* matrix was processed separately and scaled using z-score (*S_frac_*,1) to zero mean and one standard deviation (taken from MATLAB statistical toolbox). This is to ensure that all sensor responses were commensurate and no particular sensor dominates the results. An unsupervised multivariate exploratory data analysis technique such as PCA was identified as a suitable method to visualize patterns in the data, especially when the sensors are highly correlated [36]. This technique transforms a set of correlated sensors into a new set of uncorrelated sensors in a linear combination in which the amount of largest possible variance from all the sensors are presented in a decreasing order [36,37].

Each individual modality was projected separately by PCA based on the correlation matrix. An adequate number of dimensions projected by PCA were determined based on principal components (PCs) that have achieved cumulative variance of 80% or more. Further analyses to evaluate and classify those 18 different classes were performed using LDA. Cross-validation using the leave-one-out method was applied and variable selection was accomplished using Wilks’ lambda test to select the most significant variables that contribute toward the classification. Fisher linear discriminant function was also applied in this analysis. Both PCA and LDA were governed by the linear parametric multivariate analysis (MVA).

On the other hand, the Probabilistic Neural Network (PNN) [38] was selected to evaluate the behavior of non-linear parametric MVA for further classification. PNN is a part of radial basis network that is implemented based on predominant nearest neighbor classifier. The classification factor is highly dependent on the spreads of its radial basis functions. If spread is near zero, the network acts as a nearest neighbor classifier. As spread becomes larger, the designed network takes into account several nearby vectors as part of its cluster. All PCA, PNN, and LDA calculations were computed using MATLAB 7.0 and SPSS Statistics16.0, respectively.

### 2.6. Data Fusion

Recently, there have been several attempts to combine the responses of different types of electronic sensory systems, and these were performed using data fusion. Many fusion methods are based on two modality systems and performed using low level fusion (LLF) [4,5,39]. LLF was originally introduced to mimic human decisions not only based on the phytochemicals or the chemical compounds found in the solution or volatiles but also to group the samples based on their smell and taste [5]. In this experiment, PCA and LDA were chosen to perform the low level fusion and the requirement for this method is that the sensors for both modalities must be commensurate and operate in the same dimension [40]. To ensure these datasets are standardized, this new dataset (after being combined) was scaled before performing the PCA and LDA. The same transformation has been performed to classify complex herbal solutions of different brands [5]. Cross-validation using the leave-one-out method was performed using LDA on separate e-nose and e-tongue datasets, fusion of e-nose and e-tongue and sensor fusion with feature selection (sensor selection).

In addition, PNN was chosen as a non-linear method to further verify and validate the fusion of e-nose and e-tongue data. In total, there are 90 datasets of 32 variables from 18 different honey samples. Similarly, for the e-tongue, there are 90 datasets of 7 variables from the 18 different honey samples. The e-nose and e-tongue data used for PNN training and validation consists of 36 dataset samples with 32 and 7 variables, respectively. The rest of the 54 dataset samples with 32 and 7 variables of the e-nose and e-tongue were used for testing purposes.

## 3. Results and Discussion

### 3.1. FTIR Result

**Figure 3.**
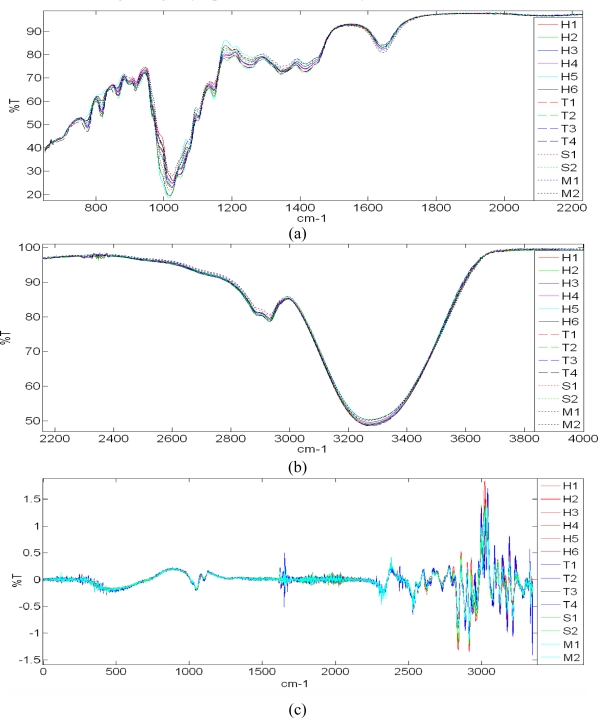
FTIR-ATR spectra of honey from different floral origin, sugar syrup and adulterated honey. (**a**) Spectral range from 550 to 2,200 cm^−1^ and (**b**) Spectral range from 2,200 to 4,000 cm^−1^. (**c**) First Order Derivatives of FTIR-ATR spectra of honeys of different floral origin, sugar syrup and adulterated honey.

FTIR spectroscopy measurements of honeys of different floral origin, syrup and adulterated samples are shown in Figure 3. These 18 different samples show high similarity and have similar spectral features. There are however some distinctive features between different floral origin, sugar syrup and adulterated honey, as shown in Figure 3(c). The spectral region from 750 to 1,500 cm^−1^ corresponds to the attenuation or absorption region of carbohydrate chains such as monosaccharides and disaccharides of honey and sugar. While the negative peak observed in between 750 and 900 cm^−1^ region shows the presence of saccharide chain and the sharp declination curved around 1,100 cm^−1^ corresponds to the C-O bond in the C-OH group [13]. Both honey and sugar samples exhibit similar characteristic in the IR spectral response due to the presence of major components in both samples. Thus, differentiation of adulterated honey with lower sugar syrup concentration is not possible and these samples are hardly screened using the FTIR method.

### 3.2. GC-MS Results

**Figure 4.**
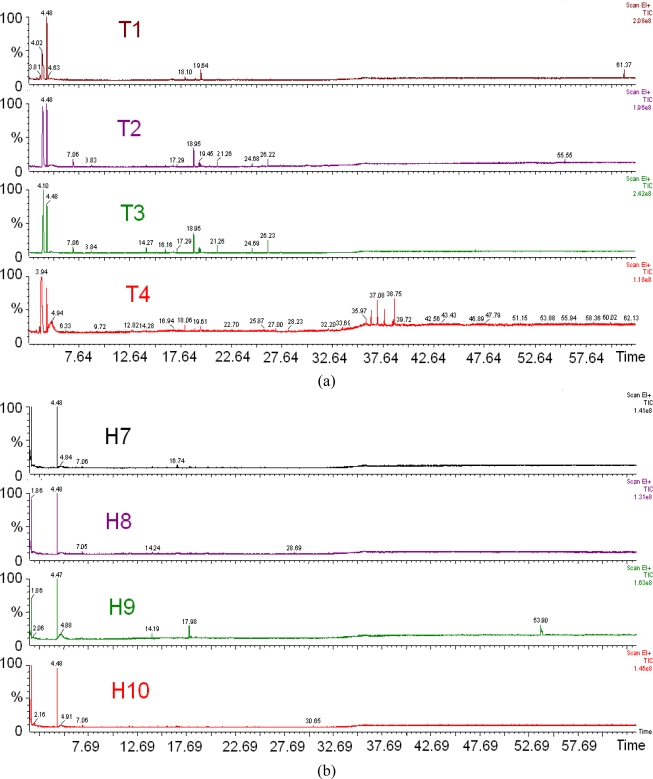

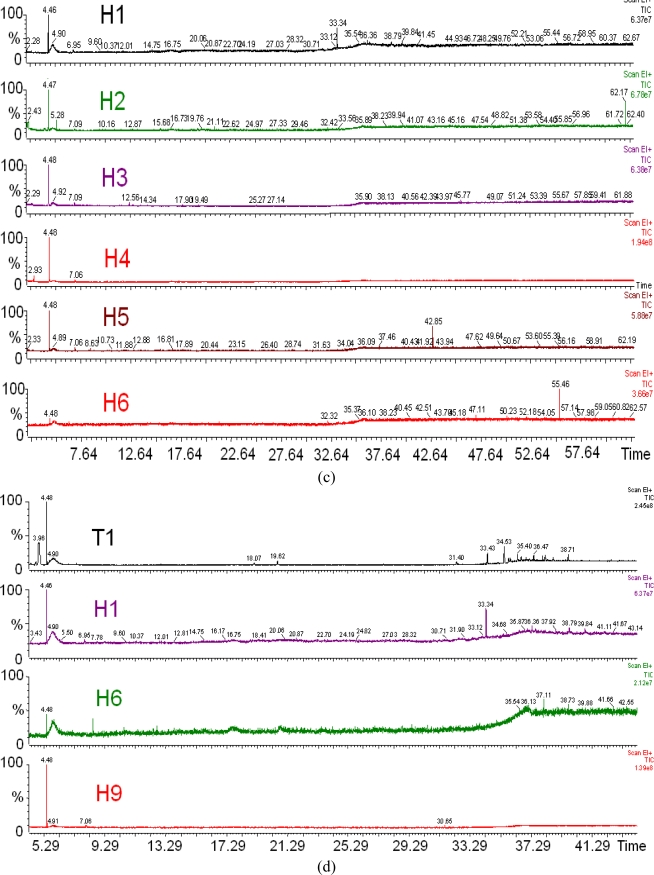

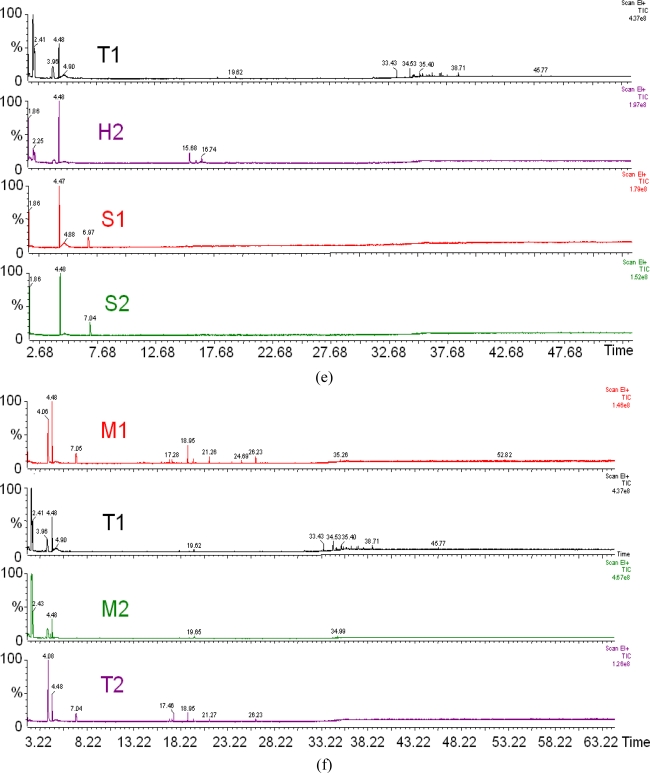
The chromatograph results of the selected monofloral honeys, polyfloral honeys, sugar and adulterated samples: (**a**) Four different brands of Tualang Honey, (**b**) Four different monofloral Honey from New Zealand, (**c**) Six different monofloral honey from Malaysia, (**d**) Comparison between monofloral and polyfloral honey, (**e**) Comparison between polyfloral and monofloral honey samples with two different brands sugar syrup, (**f**) Comparison between adulterated sample and Tualang honey.

The chromatogram plots of selected honey, sugar and adulterated samples are displayed in Figure 4. Several hundred volatile components were discovered in the chromatograms; with more than 30 distinctive peaks being present (the full component list is not shown). Fewer peaks were observed in monofloral honeys compared to polyfloral honeys, as shown in Figures 4(a–c).

At this point, it is also possible to differentiate between honeys of different floral origin and sugar concentrate. Figure 4(d) displays a distinct different peak response of honey samples of different floral origin, monofloral honeys and sugar concentrate. In addition to that, the chromatograms of the sugar syrups exhibit even fewer peaks, as shown in Figure 4(e).

However, when honey samples were mixed with sugar syrup, it is still not possible to discriminate between adulterated samples and original honey samples based on the chromatograms, as shown in Figure 4(f). The GC instrument only looks at the volatile samples and does not perceive smell as humans do. On the other hand, e-nose and e-tongue systems comprise sensor arrays that are partially selective, have the advantage and is one step closer than this analytical instrument since they can perceive the distinct smell and taste of honey.

### 3.3. Biochemical Results

The measured values of biochemical properties of honeys of different floral origin, sugar and adulterated samples are shown in Table 5. The pH value of each sample falls within the acceptable range for honey [41–44]. Unfortunately, there are no significant differences of brix, Refractive Index and pH level between honey, sugar and adulterated samples. The biochemical properties of honey were also found to vary according to geographical region [45] and these measurements were inconclusive to discriminate the adulterated honey sample. However, [23] found that although the correlations between EC-pH level and honey dilution were enough to discriminate between honeys of different floral origin, they were still unable to discriminate between adulterated samples and pure honey. The extension of this concept is somewhat similar to that proposed in this paper where the combination of e-nose and e-tongue system can be used to further discriminate adulterated samples.

### 3.4. E-Nose Results

**Figure 5.**
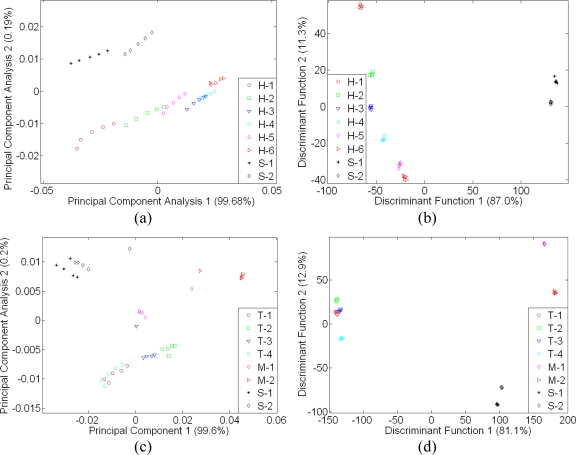

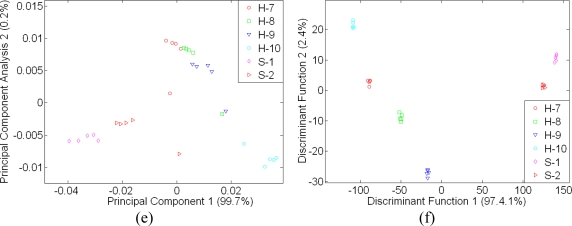
Separate plot of 32 e-nose sensor results for honey assessment: (**a**) PCA plot of Leaf, Durian, Malaluka, Coconut, Starfruit, Wax Apple Honey, Nona and Bunga Raya, (**b**) LDA plot of Leaf, Durian, Malaluka, Coconut, Starfruit, Wax Apple Honey, Nona and Bunga Raya, (**c**) PCA plot of four different brands of Tualang Honey, Nona, Bunga Raya and two adulterated samples, (**d**) LDA plot of four different brands of Tualang Honey, Nona, Bunga Raya and two adulterated samples, (**e**) PCA plot of four different floral origin of New Zealand honey, Nona and Bunga Raya, and (**f**) LDA plot of New Zealand honey of four different floral origins, Nona and Bunga Raya.

Several articles reveal that e-nose technology with an optimised pattern recognition technique can be useful in agriculture applications [33,46–47]. Figure 5 shows that LDA is much more powerful compared to PCA. The LDA technique is able to reduce drifting effects observed in the e-nose response. It is a supervised pattern classification method and is based on the determination of linear discriminant functions aim to maximize separation between groups in which within-group variance is minimized. A very distinct separation and good clustering within the sample class was observed using LDA technique in Figure 5(b,d,f), compared to PCA technique shown by Figure 5(a,c,e). In Figure 5(b), the LDA plot shows that syrup was successfully clustered and separated from monofloral honey samples, while in Figure 5(d), the LDA result is consistent with the result shown in Figure 5(b), where sugar and adulterated sample are clustered and isolated in the right-hand region. Meanwhile, the Tualang honey samples from different brands are located on the left region. Similar behaviour was also observed in Figure 5(f), where New Zealand honey varieties were also located on the left region and the syrup samples on the right.

### 3.5. E-Tongue Results

**Figure 6.**
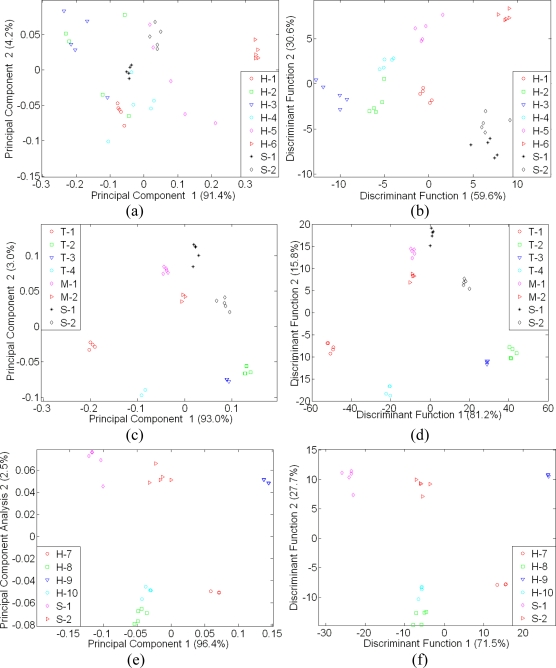
Separate plot of 7 e-tongue sensors on honey assessment: (**a**) PCA plot of Leaf, Durian, Malaluka, Coconut, Starfruit, Wax Apple Honey, Nona and Bunga Raya, (**b**) LDA plot of Leaf, Durian, Malaluka, Coconut, Starfruit, Wax Apple Honey, Nona and Bunga Raya, (**c**) PCA plot of four different brands of Tualang Honey, Nona, Bunga Raya and two adulterated samples, (**d**) LDA plot of four different brands of Tualang Honey, Nona, Bunga Raya and two adulterated samples, (**e**) PCA plot of four different floral origin of New Zealand honey, Nona and Bunga Raya and (**f**) LDA plot of four different floral origin of New Zealand honey, Nona and Bunga Raya.

Unlike the e-nose results, both PCA and LDA results of e-tongue analysis were less effective. This is due to the fact that honey and sugar are made up mostly of carbohydrate chains. Like e-nose analysis, discrimination and classification using LDA was better compared to PCA. The comparison between PCA and LDA performance is shown in Figure 6. Figure 6(b,d,f) shows better clustering behaviour and separation between the honey samples, sugar and adulterated samples. The separation between each class was very distinctive compared to the PCA displayed in Figure 6(a,c,e). In addition, the PCA was not only incapable of discriminating honey samples of different floral origin but also unable to discriminate honey from syrup samples. This can be clearly observed in Figure 6(a).

**Figure 7.**
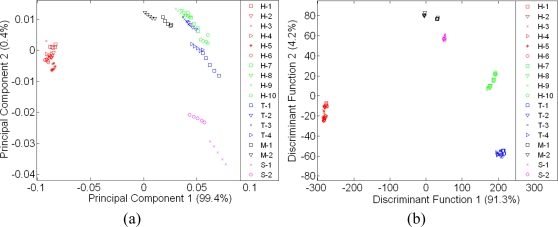
A global map of e-nose assessment on of 18 different honey, sugar and adulterated sample (**a**) Using PCA and (**b**) Using LDA.

**Figure 8.**
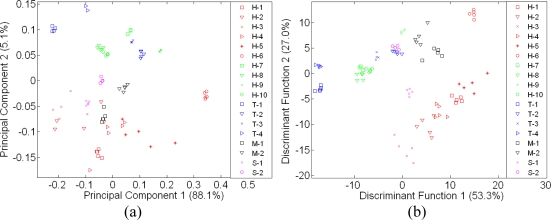
A global map of e-tongue assessment on of 18 different honey, sugar and adulterated sample (**a**) Using PCA and (**b**) Using LDA.

Although both e-nose and e-tongue results using the LDA technique seem to be very promising and can discriminate between pure honeys of different floral origin, sugar syrup and adulterated samples, this is only possible when compared with the limited number of a sample group in a particular map. As shown in Figures 7 and 8, neither PCA nor LDA were able to discriminate and distinguish honey samples of different floral origin from syrup and adulterated sample when those 18 samples were combined in one classification analysis.

In Figure 7(a), adulterated samples are hardly discriminated from pure honey samples, while in Figure 7(b), the LDA technique shows better response where the separation of sugar and adulterated samples are distinct. However, Tualang honey of different brands, a variety of honey from different floral origin and geographical origin, sugar and adulterated sample were clustered into six different classes instead of 18 different classes.

A similar response was observed by e-tongue assessment as seen in Figure 8(a,b). Both PCA and LDA were unable to discriminate the different varieties of honey, syrup and adulterated samples. The LDA technique was also found to perform better than PCA and thus, based on all the above results, PCA is found to be ineffective and a weak technique to discriminate the complex odours and taste of different honey varieties, sugar and adulterated samples. The same perception applies for human when using only smell or taste to discriminate different varieties of food. They also require more samples and training to give better discrimination and classification.

### 3.6. Human Mimicking Sensor Fusion

**Figure 9.**
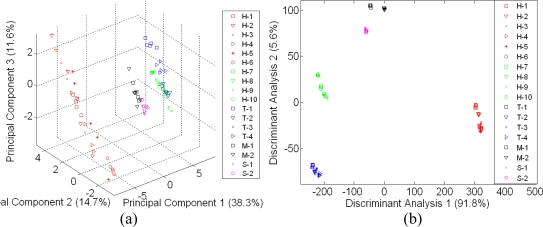
A global map of e-tongue assessment on of 18 different honey, sugar and adulterated samples (**a**) Using PCA and (**b**) Using LDA.

An improvement was observed when the data from e-nose and e-tongue were fused. Similarly, this concept applies to human senses as well. With the combination of smell and taste, individuals can discriminate food and beverages even better [48]. The discrimination and classification can be made better when we have more information, especially when this information complements each other. Figure 9(a) shows the PCA technique that was previously found to be ineffective before it is now improved. When both sensor arrays from different modalities were fused, all 39 sensors will ‘interact with each other’ and contribute towards a better classification performance [5,6]. This is clearly seen when comparing Figure 9 with Figures 7 and 8. Both sugar syrup samples were clustered as one group since they are both derived from sugar cane.

A similar behavior was observed in the Tualang honey cluster where all four different Tualang honey brands fall into one cluster. As shown previously in Figures 7(b) and 8(b), the New Zealand honey varieties were clustered as one. When fusion was performed, the classification of New Zealand honey was also improved. The improvement was also observed for the monofloral Malaysian honey.

The performance of LDA using leave-one-out approach recorded 100% correct classification of the original group cases and even after being cross-validated when sensor fusion was applied as shown in Table 6. Similar correct classification rate was also observed using sensor fusion with features selection (sensor selection). No information loss was observed when this method was applied.

Furthermore, an improvement was also observed when using the PNN classifier. The PNN classification results of separate e-nose, e-tongue, and fusion of both e-nose and e-tongue (before and after sensor selection) is shown in Table 7. The highest classification score (94.44%) was observed when applying sensor fusion with features selection and spread size of 0.001.

Honey group sample H-2 was misclassified for group H-3 and H-4, while the rest of the groups were 100% correctly classified as shown in Table 8. Sensors 2, 5 and 7 from the e-tongue were removed based on Wilks’ lambda test as shown in Table 9.

## 4. Conclusions

The brix, refractometer and pH measurements were unable to discriminate the different varieties of honey samples from syrup and adulterated samples, as the measurements show no distinct readings between the samples. The variations in both brix and pH measurements are primarily due to honey from different origins, being affected by the climate, surrounding and time of harvest. Thus, these measurements were inconclusive and cannot be used to differentiate honey of different floral and geographical origins. They also cannot be used to differentiate honey from sugar syrups.

On the contrary, GC-MS analyses using a headspace sampler method have shown that honeys of different floral origin can be successfully discriminated based on the number of peaks at different retention times observed in the chromatograms. The chromatograms of polyfloral honey such as Tualang Honey have the most number of peaks, followed by monofloral honey and syrup. Different types of polyfloral and monofloral honeys can be further differentiated and classified based on peaks observed at different retention times. For sugar syrup, only four dominant peaks were observed in the chromatograms. Both brands of sugar syrup exhibited peaks at the same retention times.

However when both sugar and honey samples were mixed together, the discrimination of adulterated samples from pure honey based on the peak and retention time was not possible. This is because the sugar syrup volatile compounds are a subset of the honey volatiles. The adulterated honey samples have both major volatiles from the carbohydrate chains, as well as flavours and aromas.

Unlike GC-MS, the e-nose system does not require very high temperature to break-up those volatile compounds into ions. E-nose conducting polymer sensors work at room temperature and react on the volatiles when they are still in a molecular form. This enables an e-nose system to perceive smell and mimic the human sensory system. The classification of different honey varieties, sugar syrups and adulterated samples were improved when LDA was employed compared to the PCA technique. However when all samples were combined in one classification analysis, LDA were inadequate to classify each sample into 18 different classes. A similar behaviour was observed when using the e-tongue to plot all samples in one classification analysis. On the other hand, the PNN classifier can successfully classify all 18 different samples (including pure honey, adulterated and pure sugar samples). The highest PNN classification using the e-nose and e-tongue is 92.59 and 90.74, respectively.

By applying the sensor fusion technique, the discrimination and classification of honey of different floral origin, sugar syrup and adulterated samples using LDA were greatly improved. The fusion using PCA was also improved. The use of a sensor fusion technique with the LDA for the e-nose and e-tongue has enabled honey of different floral origin, sugar syrup and adulterated samples to be grouped separately. However, only ten distinct groupings were observed. These ten groupings could also be associated with human preferences as it conveys the internal biochemistry and external parameter of aroma and flavour characteristic. Similarly, an improvement was also observed when using the non-linear PNN classifier. Since the nature of the data from the e-nose and e-tongue are non-linear, the PNN classifier has performed better than both linear parametric PCA and LDA technique. Single modality assessment of e-nose and e-tongue were found to be ineffective to discriminate or to classify 18 different samples used in this experiment. Thus, this technique can extend the capability of both sensors when fused together to evaluate and classify the honey samples. This is somewhat similar to the human sensory system where the fusion of these artificial sensory systems partially emulates the way human perceive the flavours and aromas of food.

In summary, by applying data fusion, the combined e-nose and e-tongue responses essentially mimic the human preference as both interact and complement each other. Hence, this fusion method has strong potential to assist human panels in making decisions, for application in honey quality assessments, including detection of adulterated samples and classification of floral as well as geographical origins. More modalities can be added in the near future such as colour and viscosity to provide additional parameters towards the realization of bio-mimicking sensor for quality assessments of a broad range of honey.

## Figures and Tables

**Table 1.** Description of different samples variety of honey and syrup used in the experiments.

**Group**	**Descriptions**	**Country of Origin**	**Type**	**Volume (V)**
H-1	Leaf Honey	Malaysia	Monofloral	5 mL
H-2	Durian Honey	Malaysia	Monofloral	5 mL
H-3	Malaluka Honey	Malaysia	Monofloral	5 mL
H-4	Coconut Honey	Malaysia	Monofloral	5 mL
H-5	Starfruit Honey	Malaysia	Monofloral	5 mL
H-6	Wax apple honey	Malaysia	Monofloral	5 mL
H-7	Rewarewa Honey	New Zealand	Monofloral	5 mL
H-8	Kamahi Honey	New Zealand	Monofloral	5 mL
H-9	Blue Borage Honey	New Zealand	Monofloral	5 mL
H-10	Wild Flower Honey	New Zealand	Monofloral	5 mL
T-1	Tualang Honey (3 Lebah)	Malaysia	Polyfloral	5 mL
T-2	Tualang Honey (Al-Syifa)	Malaysia	Polyfloral	5 mL
T-3	Tualang Honey (RoseBee)	Malaysia	Polyfloral	5 mL
T-4	Tualang Honey (Agro Mas)	Malaysia	Polyfloral	5 mL
S-1	Sugar Syrup (Nona)	Malaysia	Sugarcane Syrup	5 mL
S-2	Sugar Syrup (Bunga Raya)	Malaysia	Sugarcane Syrup	5 mL
M-1	Tualang Honey (Al-Shifa) + Syrup (Nona)	Malaysia	Adulterated Tualang Honey	3 mL:2 mL
M-2	Tualang Honey (3 Lebah) + Syrup (Bunga Raya)	Malaysia	Adulterated Tualang Honey	3 mL:2 mL

**Table 2.** E-nose parameter settings for honey, syrup and adulterated samples assessment.

	**Cycle**	**Time(s)**	**Pump Speed**
	
Sampling setting	Baseline Purge	10	120 mL/min
	Sample Draw	30	120 mL/min
	Idle Time	3	-
	Air Intake Purge	80	160 mL/min

**Table 3.** Chalcogenide-based potentiometric electrodes used in the e-tongue.

**Sensor Label**	**Description**
Fe3+	Ion-selective sensor for Iron ions
Cd2+	Ion-selective sensor for Cadmium ions
Cu2+	Ion-selective sensor for Copper ions
Hg2+	Ion-selective sensor for Mercury ions
Ti+	Ion-selective sensor for Titanium ions
S2−	Ion-selective sensor for Sulfur ions
Cr (VI)	Ion-selective sensor for Chromium ions

HI 5311	Reference probe using Ag/AgCl electrode

**Table 5.** Brix, Refractive Index and pH level.

**Honey Sample**	**^o^Brix Level**	**Refractive Index**	**pH Level**
H-1	77.9	1.4853	3.61
H-2	80.3	1.4916	3.65
H-3	77.9	1.4853	3.77
H-4	72.8	1.4723	4.13
H-5	78.4	1.4865	3.94
H-6	81.0	1.4933	3.84
H-7	81.5	1.4946	4.13
H-8	75.5	1.4792	4.57
H-9	81.5	1.4945	3.57
H-10	77.2	1.4835	3.82
T-1	74.9	1.4776	3.38
T-2	80.8	1.4928	3.86
T-3	78.2	1.4862	3.88
T-4	72.9	1.4726	3.44
S-1	77.4	1.4839	3.35
S-2	81.8	1.4956	3.67
M-1	79.1	1.4883	3.87
M-2	69.4	1.4640	4.04

**Mean**	77.7	1.4848	3.81
**Max**	81.8	1.4956	4.57
**Min**	69.4	1.464	3.35

**Table 6.** LDA classification results using leave-one-out approaches.

**Modality**	**Original grouped cases that were correctly classified**	**Cross-validated grouped cases that were correctly classified**
**E-nose**	100%	98.9%
**E-tongue**	94.4%	96.7%
**Sensor fusion**	100%	100%
**Sensor fusion (*with features selection*)**	100%	100%

**Table 7.** PNN Classification results.

**Modality**	**Datasets**			**Spread Size of Radial Basis Function**			
				**0.1**	**0.01**	**0.001**	**0.0001**

	**Training and Validation**	**Testing**	**Number of Sensors**	**Classification Results (%)**			
**E-nose**	36	54	32	83.33	83.33	90.74	92.59
**E-tongue**	36	54	7	81.48	87.03	90.74	64.82
**Sensor fusion**	36	54	39	81.48	88.89	92.59	64.82
**Sensor fusion (*with features selection*)**	36	54	36	87.04	88.89	94.44	77.78

**Table 8.** Detailed PNN classification results on sensor fusion with features selection and spread size of 0.001.

**Honey Sample**	Predicted Group Membership																	
	**H-1**	**H-2**	**H-3**	**H-4**	**H-5**	**H-6**	**H-7**	**H-8**	**H-9**	**H-10**	**T-1**	**T-2**	**T-3**	**T-4**	**S-1**	**S-2**	**M-1**	**M-2**
**H-1**	**3**	0	0	0	0	0	0	0	0	0	0	0	0	0	0	0	0	0
**H-2**	0	0	**1**	**2**	0	0	0	0	0	0	0	0	0	0	0	0	0	0
**H-3**	0	0	**3**	0	0	0	0	0	0	0	0	0	0	0	0	0	0	0
**H-4**	0	0	0	**3**	0	0	0	0	0	0	0	0	0	0	0	0	0	0
**H-5**	0	0	0	0	**3**	0	0	0	0	0	0	0	0	0	0	0	0	0
**H-6**	0	0	0	0	0	**3**	0	0	0	0	0	0	0	0	0	0	0	0
**H-7**	0	0	0	0	0	0	**3**	0	0	0	0	0	0	0	0	0	0	0
**H-8**	0	0	0	0	0	0	0	**3**	0	0	0	0	0	0	0	0	0	0
**H-9**	0	0	0	0	0	0	0	0	**3**	0	0	0	0	0	0	0	0	0
**H-10**	0	0	0	0	0	0	0	0	0	**3**	0	0	0	0	0	0	0	0
**T-1**	0	0	0	0	0	0	0	0	0	0	**3**	0	0	0	0	0	0	0
**T-2**	0	0	0	0	0	0	0	0	0	0	0	**3**	0	0	0	0	0	0
**T-3**	0	0	0	0	0	0	0	0	0	0	0	0	**3**	0	0	0	0	0
**T-4**	0	0	0	0	0	0	0	0	0	0	0	0	0	**3**	0	0	0	0
**S-1**	0	0	0	0	0	0	0	0	0	0	0	0	0	0	**3**	0	0	0
**S-2**	0	0	0	0	0	0	0	0	0	0	0	0	0	0	0	**3**	0	0
**M-1**	0	0	0	0	0	0	0	0	0	0	0	0	0	0	0	0	**3**	0
**M-2**	0	0	0	0	0	0	0	0	0	0	0	0	0	0	0	0	0	**3**

**Table 9.** Wilks’ Lamda test.

**Modality**	**Sensor Label**	**Wilks’ Lambda**	**F**
**E-Nose**	SENSOR 01	0.006	752.572
	SENSOR 02	0.002	2057.926
	SENSOR 03	0.003	1363.984
	SENSOR 04	0.001	2926.130
	SENSOR 05	0.021	196.178
	SENSOR 06	0.019	217.367
	SENSOR07	0.009	453.653
	SENSOR 08	0.006	707.176
	SENSOR 09	0.007	570.551
	SENSOR 10	0.018	236.565
	SENSOR 11	0.003	1642.171
	SENSOR 12	0.004	1068.317
	SENSOR 13	0.014	298.223
	SENSOR 14	0.006	714.845
	SENSOR 15	0.006	722.176
	SENSOR 16	0.013	316.414
	SENSOR 17	0.021	194.726
	SENSOR 18	0.009	457.123
	SENSOR 19	0.003	1534.570
	SENSOR 20	0.016	259.070
	SENSOR 21	0.015	287.756
	SENSOR 22	0.020	210.748
	SENSOR 23	0.008	498.394
	SENSOR 24	0.001	3227.972
	SENSOR 25	0.006	745.076
	SENSOR 26	0.021	197.183
	SENSOR 27	0.005	853.482
	SENSOR 28	0.009	489.800
	SENSOR 29	0.006	660.677
	SENSOR 30	0.014	301.869
	SENSOR 31	0.004	949.522
	SENSOR 32	0.010	427.409

**E-Tongue**	SENSOR 1	0.034	119.304
	SENSOR 2	0.834	0.842
	SENSOR 3	0.026	159.871
	SENSOR 4	0.092	41.619
	SENSOR 5	0.594	2.897
	SENSOR 6	0.042	96.310
	SENSOR 7	0.712	1.713
